# C3a/C3aR synergies with TGF-β to promote epithelial-mesenchymal transition of renal tubular epithelial cells via the activation of the NLRP3 inflammasome

**DOI:** 10.1186/s12967-023-04764-6

**Published:** 2023-12-11

**Authors:** Danyu You, Kun Nie, Xiaoting Wu, Mengjie Weng, Liyan Yang, Yi Chen, Jiong Cui, Jianxin Wan

**Affiliations:** 1 Department of Nephrology, Blood Purification Research Center, The First Affiliated Hospital of Fujian Medical University, Chazhong Road 20, Fuzhou, 350005 China; 2 Fujian Clinical Research Center for Metabolic Chronic Kidney Disease, The First Affiliated Hospital of Fujian Medical University, Fuzhou, 350005 China; 3 Department of Nephrology, National Regional Medical Center, Binhai Campus of the First Affiliated Hospital, Fujian Medical University, Fuzhou, 350212 China

**Keywords:** Complement component 3a receptor, The NLRP3 inflammasome, Epithelial-mesenchymal transition

## Abstract

**Background:**

Complement component 3a and its receptor (C3a/C3aR) and the nucleotide-binding oligomerization domain-like receptor protein-3 (NLRP3) inflammasome contribute to epithelial-mesenchymal transition (EMT). However, the relationship between C3a/C3aR and the NLRP3 inflammasome in EMT remains unclear. This study aimed to elucidate the roles of C3a/C3aR and the NLRP3 inflammasome involved in TGF-β-induced EMT.

**Method:**

Mouse renal tubular epithelial cells (TCMK-1) were exposed to C3a and TGF-β for 48 h. C3aR antagonist, MCC950, an inhibitor of the NLRP3 inflammasome and PD98059, an inhibitor of ERK signaling, were respectively applied to pretreat the cells at 30 min before C3a and TGF-β administration.The cells were collected for western blot, immunofluorescence staining and ELISA. Unilateral ureteral obstruction (UUO) models were established using male C57BL/6 wild-type (WT) mice and age-matched C3aR-deficient mice. MCC950 was intraperitoneally injected in UUO mice. Kidney samples were collected for immunohistochemistry staining.

**Results:**

In vitro, C3a synergized with TGF-β to promote EMT and the activation of the NLRP3 inflammasome. Inhibition of C3aR attenuated EMT and the activation of the NLRP3 inflammasome. Inhibition of the NLRP3 inflammasome alleviated EMT but didn’t affect the expression of C3aR. Inhibition of ERK signaling inhibited the activation of the NLRP3 inflammasome. In vivo, the expression of IL-1β was significantly higher in UUO mice compared to the sham-operated mice. C3aR deficiency and inhibition of the NLRP3 Inflammasome contributed to decreased IL-1β in UUO mice.

**Conclusion:**

Our data revealed that C3a/C3aR synergies with TGF-β to activate the NLRP3 inflammasome to promote epithelial-mesenchymal transition of renal tubular epithelial cells through ERK signaling, and the way in which C3aR activates the inflammasome is to promote the assembly of the NLRP3 inflammasome.

## Background

Chronic kidney disease (CKD), on account of its high morbidity and mortality, has become a global health burden. Globally, the prevalence of CKD was 9.1% and 1.2 million people died from CKD in 2017 [1]. In China, the prevalence in adults was 8.2% in 2019 [2]. Renal fibrosis is the most important pathological process and the final pathway to CKD, regardless of the initial etiology [3]. Epithelial-mesenchymal transition (EMT) is a biological process associated with fibrosis, involving the transdifferentiation of polarized epithelial cells into mesenchymal cells [4]. Thus the alleviation of EMT of renal tubular epithelial cells (RTECs) can effectively ameliorate renal fibrosis, resulting in decelerating the progression of CKD.

Complement C3 is a crucial component of the complement system which contributes to a variety of tissue injuries. The smaller fragment of C3, known as C3a, has been proved to play a vital role in the pathogenesis of several kidney diseases by binding to its receptor (C3aR) [5]. Our previous study demonstrated that C3a induces EMT of RTECs [6, 7]. In proteinuric nephropathy, EMT is mediated by the interaction between C3a and C3aR, whereas deficiency of C3aR leads to less tubulointerstitial fibrosis [8].

The nucleotide-binding oligomerization domain-like receptor protein-3 inflammasome (NLRP3 inflammasome) is a protein oligomeric complex composed of NLRP3, apoptosis associated speck-like protein (ASC), and pro-Caspase-1. Once the NLRP3 inflammasome is activated, pro-Caspase-1 undergoes cleavage to form active Caspase-1.This active Caspase-1 then regulates the maturation of IL-1β and IL-18, leading to inflammation and fibrosis [9]. Additionally, the activation of the NLRP3 inflammasome has also been demonstrated to induce EMT [10]. In NRK-52E cells, the activation of the NLRP3 inflammasome induced by TGF-β promotes EMT [11].

Although both C3a/C3aR and the NLRP3 inflammasome contribute to EMT, the relationship between C3a/C3aR and the NLRP3 inflammasome in EMT remains unclear. Recently, we proposed that C3aR aggravated renal interstitial fibrosis by regulating the activation of the NLRP3 inflammasome (particularly regulating the assembly of the inflammasome) in the unilateral ureteral obstruction (UUO) model [12]. We also found that C3aR and NLRP3 were both expressed in RTECs of UUO mice [12]. To further validate this recent perspective in vitro, we investigated the relationship between C3a/C3aR and the NLRP3 inflammasome in EMT of RTECs in this study. Our hypothesis posited that C3a/C3aR modulates the activation of the NLRP3 inflammasome to facilitate EMT of RTECs.

## Methods and materials

### Cell culture and treatments

Mouse renal tubular epithelial cells (TCMK-1, ATCC®, CCL-139™) were purchased from American type culture collection (Manassas, USA) and were cultivated in MEM/EBSS medium (Gibco, USA) with 10% fetal bovine serum (FBS). 24 h prior to treatment, TCMK-1 cells were starved in MEM/EBSS medium containing 0.5% FBS, followed by exposure to 1 μg/ml C3a (8085-C3, R&D Systems, USA) and 10 ng/ml TGF-β (100-18B, PeproTech, USA) for 48 h. 20 μM C3aRa (HY-101502A, MedChemExpress, USA), 10 μM MCC950 (HY-12815, MedChemExpress, USA) and 20 μM PD98059 (HY-12028, MedChemExpress, USA) were respectively applied to pretreat TCMK-1 cells at 30 min before C3a and TGF-β administration.

### Western blot

Proteins from cultured TCMK-1 cells were extracted using RIPA buffer containing protease. Protein concentrations were quantified using a BCA protein assay kit. The protein samples were separated by 6–15% sodium dodecyl sulfate–polyacrylamide gel electrophoresis and transferred to a polyvinylidene difluoride membrane, followed by blocking with 5% skim milk for 1 h. Then, the membranes were incubated overnight at 4 °C with the following antibodies: C3aR (1:500, sc-133272, Santacruz, USA), NLRP3 (1:2000, ab263899, Abcam, USA), Caspase 1–20 (1:1000, AG-20B-0042, Adipogen, USA), p-ERK1/2 (1: 1000, #4370, Cell Signaling Technology, USA), ERK1/2 (1: 2000, #4695, Cell Signaling Technology, USA), and β-tubμlin (1:5000, P07437, Abways, China) as an internal control. Next, the membranes were incubated with appropriate secondary antibodies for 1 h at room temperature. Specific bands were detected by enhanced chemiluminescence.

### Immunofluorescence

The medium was removed and the cells were fixed for 40 min with 4% paraformaldehyde. Then the cells were stained according to standard immunofluorescence procedures. After incubation with anti-α-SMA (1:500, ab32575, Abcam, USA) and anti-ZO-1(1:100, ab221547, Abcam, USA) antibodies at 4 °C overnight, appropriate Alexa Fluor 488 secondary antibodies (1:200, ZSJQ-BIO, China) were used for fluorescent labeling. The nuclei were stained with DAPI, and images were obtained using a fluorescence microscope (Ts2R-FL, Nikon, Japan). Image analysis was performed in ImageJ software.

### ELISA

Protein level of IL-1β in supernatants of cell cultures were detected by ELISA Kits (EMC001b, NeoBioscience, China) according to the manufacturer’s instructions. The OD was measured by a multimode ELISA plate reader (Multiskan FC, ThermoFisher, USA).

### Animals

The total number of mice used in the study was 60 and the number used in each group was 6. All animal protocols were reviewed and approved by the Institutional Animal Care and Use Committee of Fujian Medical University (Approval Number: 2021–0529). The health status of the mice was monitored everyday. In the event of inter-mouse aggression, it was necessary to house them in separate cages. Euthanasia was conducted if the mice exhibited loss of tactile response, immobility of the eyes, severe weight loss (20% of body weight), dehydration (fur and eyes sinking), and other more severe abnormal behaviors. C57BL/6 mice with C3aR KO (C3aR KO mice; strain B6/JGpt-C3ar1em6CD4816/Gpt) and without C3aR KO [C57BL/6 wild-type (WT) mice] were purchased from GemPharmatech Co., Ltd. (China). CRISPR/Cas9 technology was used to knockout C3aR gene and generate chimera F0 mice. Positive F0 mice confirmed by PCR genotyping and sequence analysis were mated with wild-type C57BL/6 mice to generate F1 heterozygous mice. Positive F1 mice confirmed by PCR genotyping and sequence analysis were mated with each other to generate C3aR-KO homozygotes (C3aR KO mice). Animals were housed in a pathogen-free, temperature-controlled environment with a 12 h/12 h light/dark photocycle and had ad libitum access to food and tap water to avoid dehydration-induced hypovolemia. Male C3aR KO mice and age-matched C57BL/6 WT mice (8–12 weeks old) were used for experiments. Mice were anesthetized with ketamine (50 mg/kg body weight) and xylazine (100 mg/kg body weight), and then, a lateral incision on the back was created. UUO was achieved by double ligation of the renal pelvis and the upper 1/3 of the left ureter; this was followed by making a cut in the middle of the double ligation. Sham-operated mice, which underwent an identical procedure but without ureteric ligation, were used as sham control. Mice were euthanized at day 14 after the operation, and obstructed kidneys were collected.

### Inhibition of the NLRP3 inflammasome in mice

The NLRP3 inflammasome was inhibited in vivo by intraperitoneally injecting 20 mg/kg MCC950 (MCE, USA, HY-12815A) in 200 µL of normal saline (NS) or vehicle (200 µL of NS) once daily from the day of operation to the day of euthanasia (UUO model).

### Immunohistochemistry staining

The kidneys were fixed with 4% paraformaldehyde and embedded in paraffin. Then, 4-μm sections were prepared for immunohistochemistry staining. The paraffin-embedded sections were blocked with 3% bovine serum albumin (Sigma Aldrich, USA) in phosphate-buffered saline (PBS). For staining involving horseradish peroxidase-conjugated secondary antibodies, endogenous peroxidase was blocked with H_2_O_2_. Sections were then incubated overnight at 4 °C with rabbit multiclonal antibodies against IL-1β (1: 500, ab283818, Abcam, USA), followed by incubation for 1 h at room temperature with appropriate secondary antibodies that were conjugated with horseradish peroxidase. Isotype-matched IgGs were used as a negative control. Immunohistological staining results were quantified using ImageJ software.

### Statistical analysis

Data are expressed as means ± SD. Normality of distribution was tested using the Shapiro–Wilk test before analysis. Comparison between groups was performed by one-way ANOVA using the least significant difference test (equal variance) or Dunnett’s T3 test (unequal variance) for *post-hoc* assessments. Two-way ANOVA was used for the experiments invloving two factors. A value of *P *< 0.05 was considered statistically significant. All statistical analyses were calculated with SPSS 25.0.

## Results

### C3a synergized with TGF-β to promote EMT

**Fig. 1:**
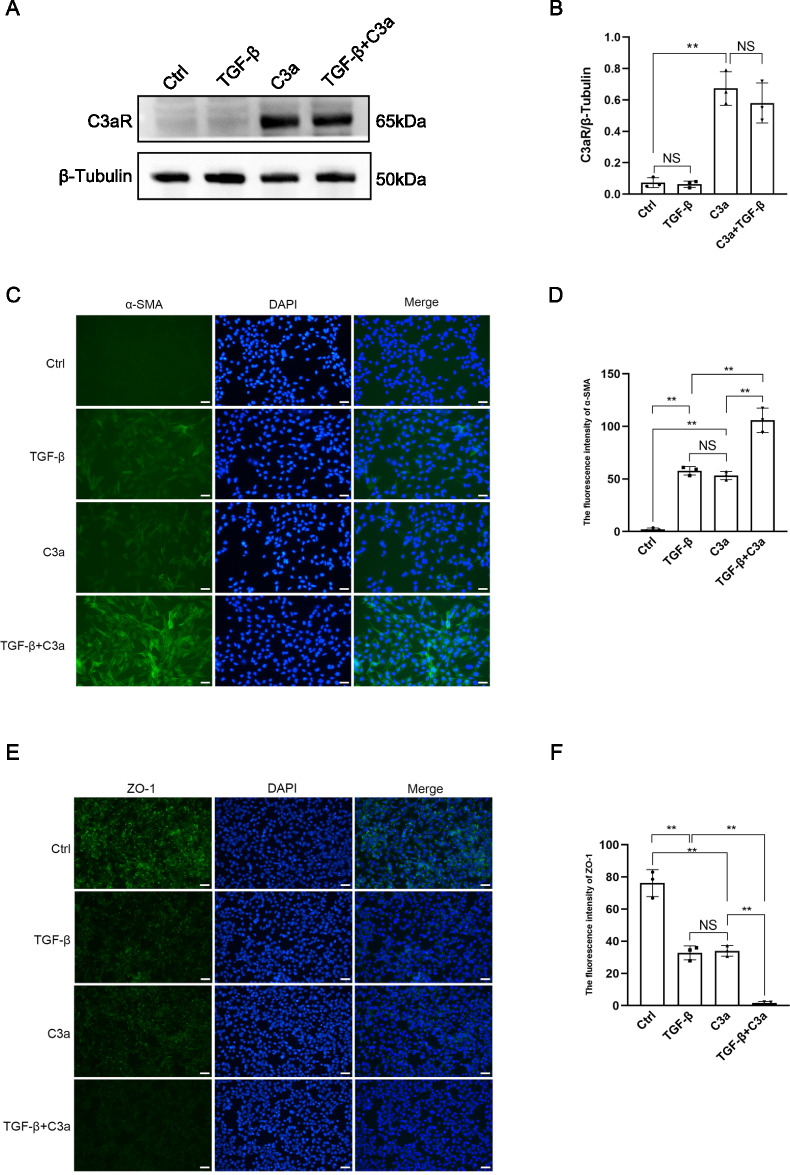
C3a synergized with TGF-β to promote EMT. **A** Western blot of C3aR of TCMK-1 cells induced by TGF-β, C3a, and C3a combined with TGF-β. **B** Quantitative analysis of C3aR. **C** Immunofluorescent staining of α-SMA of TCMK-1 cells induced by TGF-β, C3a, and C3a combined with TGF-β. **D** α-SMA-positive area. **E** Immunofluorescent staining of ZO-1 of TCMK-1 cells induced by TGF-β, C3a, and C3a combined with TGF-β. **F** ZO-1-positive area. Scale bar:50 μm.**P < 0.01;NS, No significance

Western blot showed that the expression of C3aR increased after TCMK-1 cells were stimulated by C3a and C3a combined with TGF-β, compared with the cells exposed to TGF-β alone (Fig. 1A, B). This result indicated that C3a was a C3aR agonist, while TGF-β was not. Immunofluorescent staining showed that the expression of α-SMA increased and the expression of ZO-1 decreased significantly after TCMK-1 cells were stimulated by C3a combined with TGF-β, compared with the cells exposed to C3a alone and TGF-β alone (Fig. 1C–F). This result indicated that C3a synergized with TGF-β to promote EMT of TCMK-1 cells.

### C3a synergized with TGF-β to promote the activation of the NLRP3 inflammasome

**Fig. 2:**
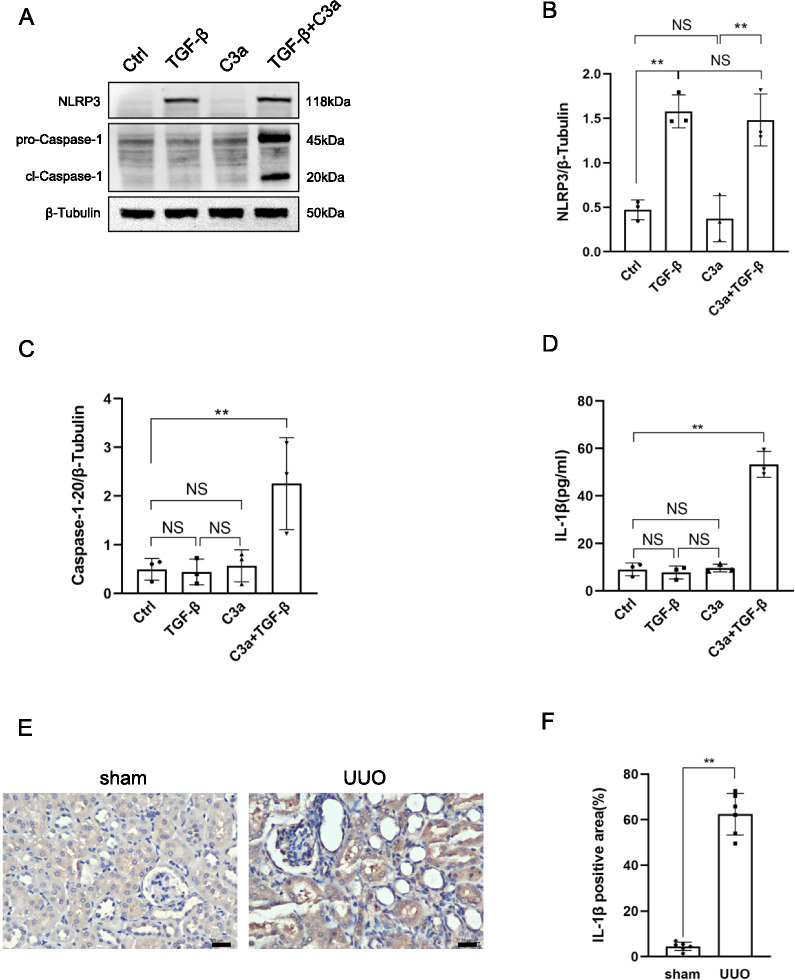
C3a synergized with TGF-β to promote the activation of the NLRP3 inflammasome.** A** Western blot of NLRP3 and Caspase-1–20 of TCMK-1 cells induced by TGF-β, C3a, and C3a combined with TGF-β. **B** Quantitative analysis of NLRP3. **C** Quantitative analysis of Caspase-1–20. **D** ELISA of IL-1β of TCMK-1 cells induced by TGF-β, C3a, and C3a combined with TGF-β. **E** IL-1β immunohistochemistry of kidney sections of WT mice (sham operation/UUO). **F** IL-1β-positive area. Scale bar:25 μm.**P < 0.01;NS, No significance

The expression of NLRP3 increased after TCMK-1 cells were stimulated by TGF-β alone, while the expression of Caspase-1–20 and the secretion of IL-1β remained unchanged. In this study, Caspase 1–20 was used as active form of Caspase-1. The expressions of NLRP3 and Caspase-1–20 and the secretion of IL-1β didn’t change after the cells were stimulated by C3a alone. However, the expressions of NLRP3 and Caspase-1–20 and the secretion of IL-1β increase significantly increased after the cells were stimulated by C3a combined with TGF-β. These findings suggested that C3a synergized with TGF-β to promote the activation of the NLRP3 inflammasome of TCMK-1 cells (Fig. 2A–D). The mice model of UUO is a kind of classical renal fibrosis model. The expression of IL-1β was significantly higher in UUO mice compared to the sham-operated mice. Immunohistochemistry also revealed that IL-1β was predominantly expressed in renal tubular epithelial cells of UUO mice (Fig. 2E, F).

### Inhibition of C3aR attenuated EMT

**Fig. 3:**
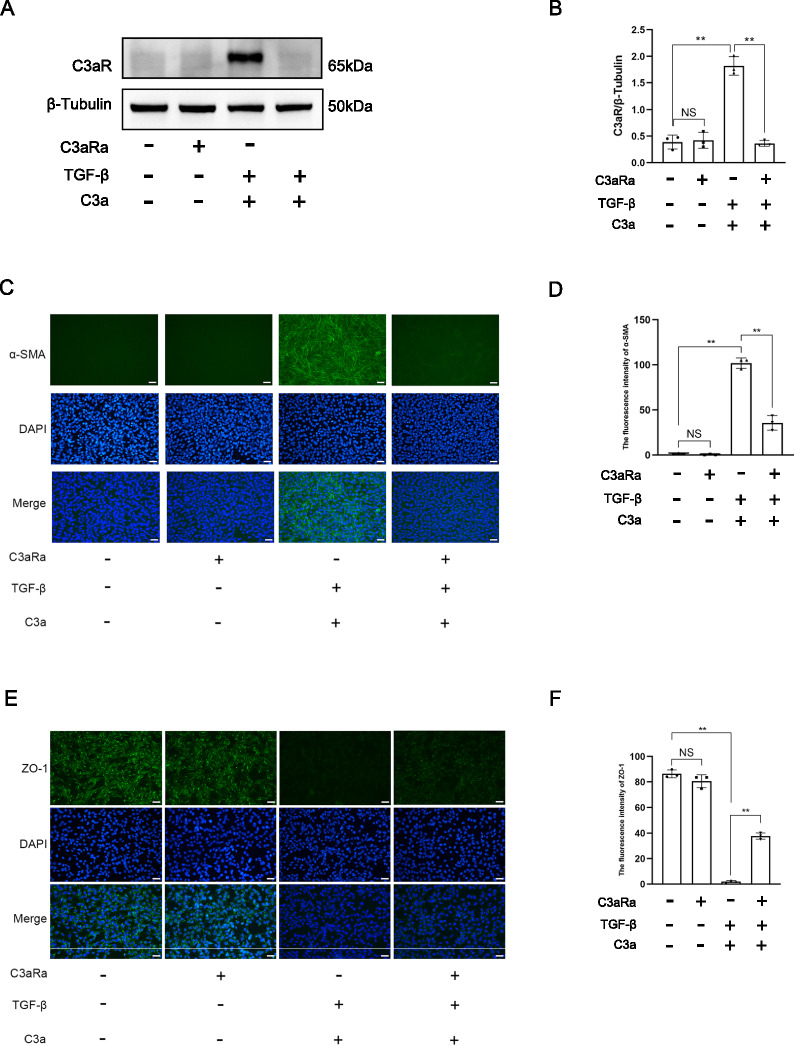
Inhibition of C3aR attenuated EMT. **A** Western blot of C3aR of TCMK-1 cells induced by C3aRa, TGF-β + C3a, and TGF-β + C3a + C3aRa. **B** Quantitative analysis of C3aR. **C** Immunofluorescent staining of α-SMA of TCMK-1 cells induced by C3aRa, TGF-β + C3a, and TGF-β + C3a + C3aRa. **D** α-SMA-positive area. **E** Immunofluorescent staining of ZO-1 of TCMK-1 cells induced by C3aRa, TGF-β + C3a, and TGF-β + C3a + C3aRa. **F** ZO-1-positive area. Scale bar:50 μm.**P < 0.01; NS, No significance

Western blot showed that C3aR antagonist (C3aRa) restrained the expression of C3aR induced by C3a combined with TGF-β in TCMK-1 cells (Fig. 3A, B). Immunofluorescent staining showed that C3aRa reduced the expression of α-SMA significantly and increased the expression of ZO-1 significantly after TCMK-1 cells were stimulated by C3a combined with TGF-β (Fig. 3C–F). These data elucidated that inhibition of C3aR attenuated EMT induced by C3a combined with TGF-β.

### Inhibition of C3aR attenuated the activation of the NLRP3 inflammasome

**Fig. 4:**
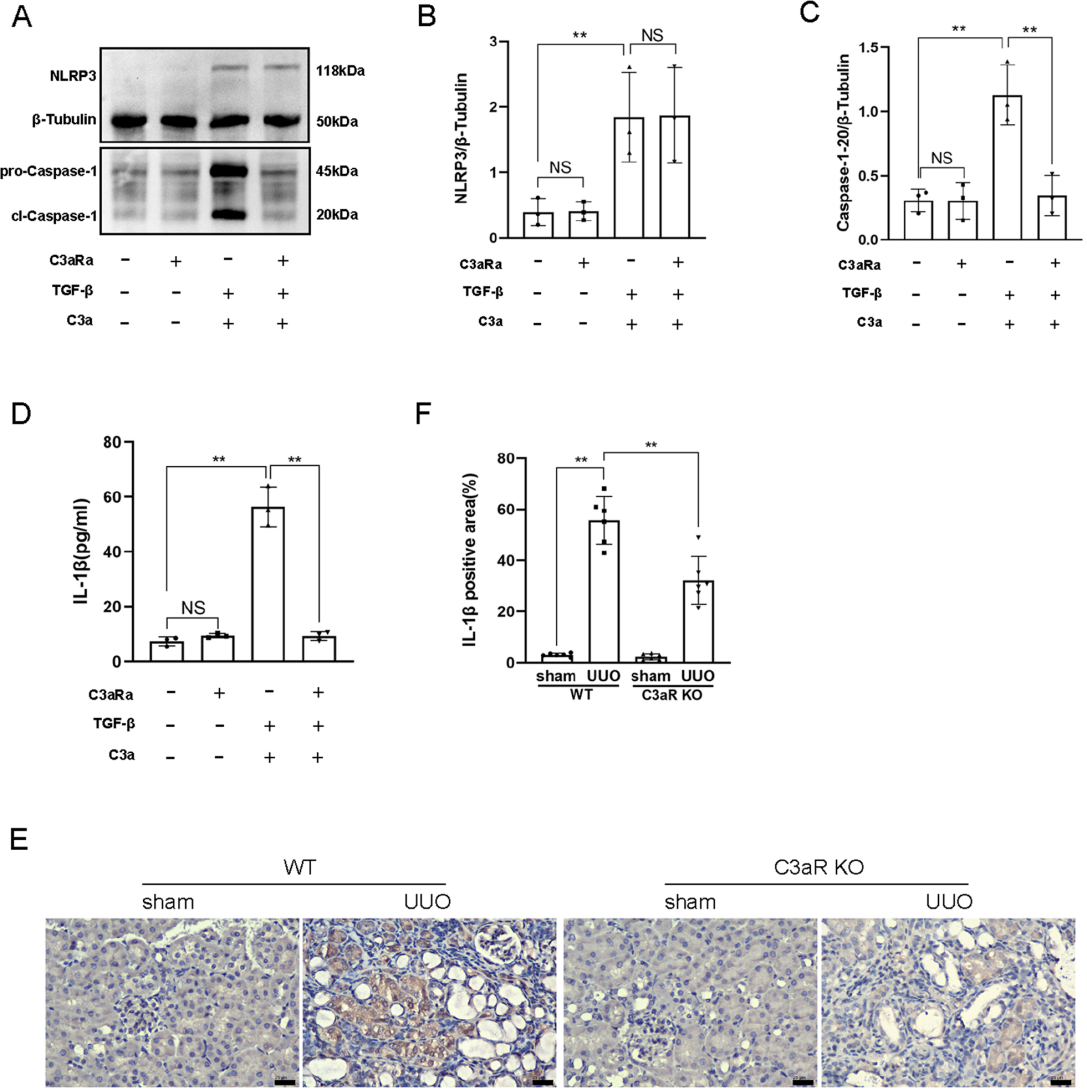
Inhibition of C3aR attenuated the activation of the NLRP3 inflammasome.** A** Western blot of NLRP3 and Caspase-1–20 of TCMK-1 cells induced by C3aRa, TGF-β + C3a, and TGF-β + C3a + C3aRa. **B** Quantitative analysis of NLRP3. **C** Quantitative analysis of Caspase-1–20. **D** ELISA of IL-1β of TCMK-1 cells induced by C3aRa, TGF-β + C3a, and TGF-β + C3a + C3aRa. **E** IL-1β immunohistochemistry of kidney sections of WT mice (sham operation/UUO) and C3aR KO mice (sham operation/UUO). **F** IL-1β-positive area. Scale bar:25 μm.**P < 0.01; NS, No significance

C3aRa didn’t reduce the expression of NLRP3 induced by C3a combined with TGF-β, but it reduced the expression of Caspase-1–20 and the secretion of IL-1β (Fig. 4A–D). This result suggested that inhibition of C3aR attenuated the activation of the NLRP3 inflammasome induced by C3a combined with TGF-β. The similar result was also proved in C3aR knock-out UUO (C3aR KO-UUO) mice. Immunohistochemistry also revealed that the expression of IL-1β decreased significantly in renal tubular epithelial cells of C3aR KO-UUO mice compared with wild-type UUO (WT-UUO) mice (Fig. 4E, F).

### Inhibition of the NLRP3 Inflammasome restrained the activation of the NLRP3 inflammasome

**Fig. 5:**
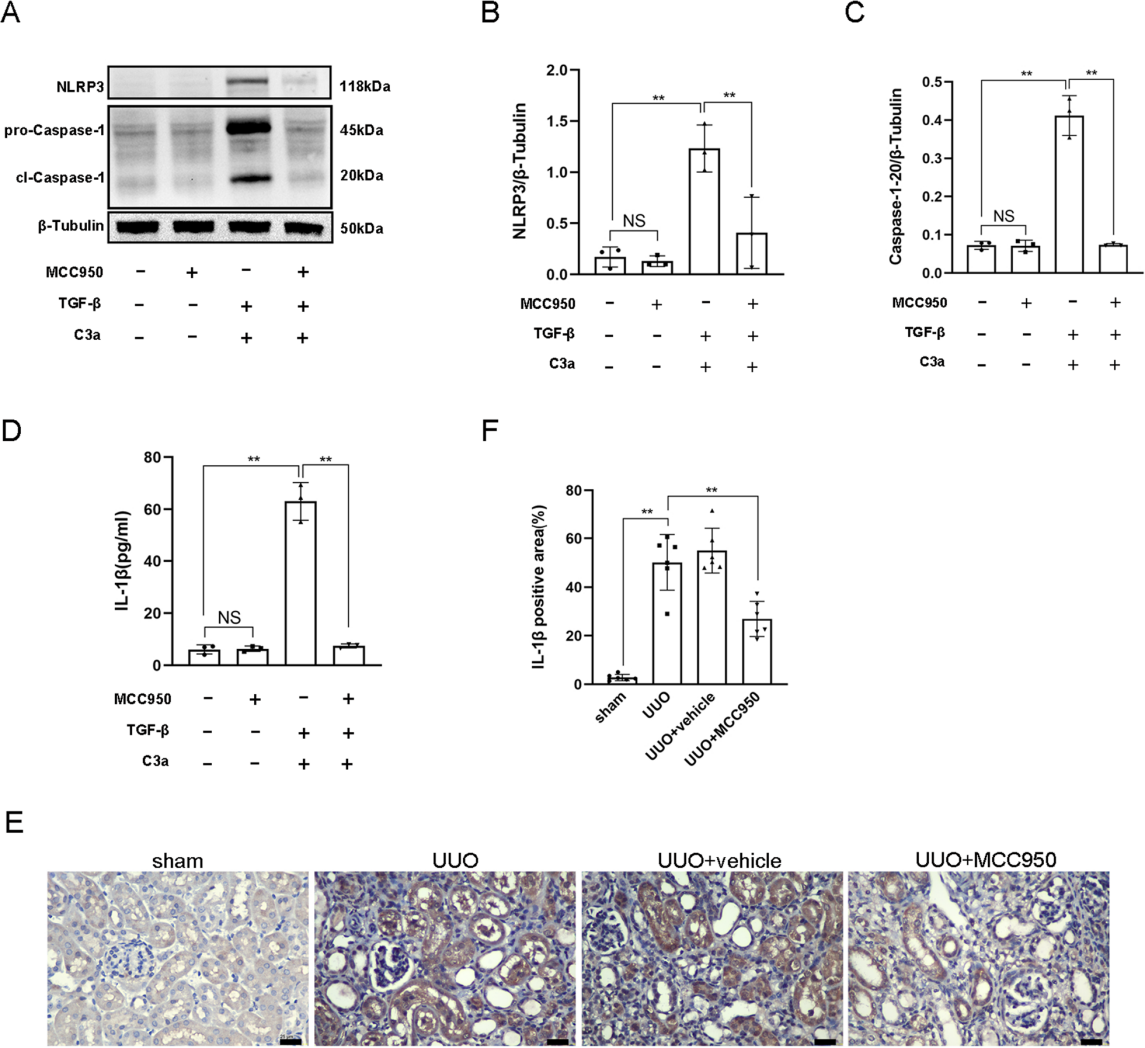
Inhibition of the NLRP3 Inflammasome restrained the activation of the NLRP3 inflammasome.** A** Western blot of NLRP3 and Caspase-1–20 of TCMK-1 cells induced by MCC950, TGF-β + C3a, and TGF-β + C3a + MCC950. **B** Quantitative analysis of NLRP3. **C** Quantitative analysis of Caspase-1–20. **D** ELISA of IL-1β of TCMK-1 cells induced by MCC950, TGF-β + C3a, and TGF-β + C3a + MCC950. **E** IL-1β immunohistochemistry of kidney sections of WT mice (sham operation, UUO, UUO + vehicle, and UUO + MCC950). **F** IL-1β-positive area. Scale bar:25 μm.**P < 0.01; NS, No significance

MCC950, an inhibitor of the NLRP3 inflammasome, suppressed the expression of NLRP3 and Caspase-1–20 and the secretion of IL-1β induced by C3a combined with TGF-β in TCMK-1 cells (Fig. 5A–D). This result demonstrated that inhibition of the NLRP3 inflammasome restrained the activation of the NLRP3 inflammasome induced by C3a combined with TGF-β. Immunohistochemistry also proved that the expression of IL-1β was downregulated in renal tubular epithelial cells after the injection of MCC950 in UUO mice (Fig. 5E, F).

### Inhibition of the NLRP3 inflammasome alleviated EMT but didn’t affect the expression of C3aR

**Fig. 6:**
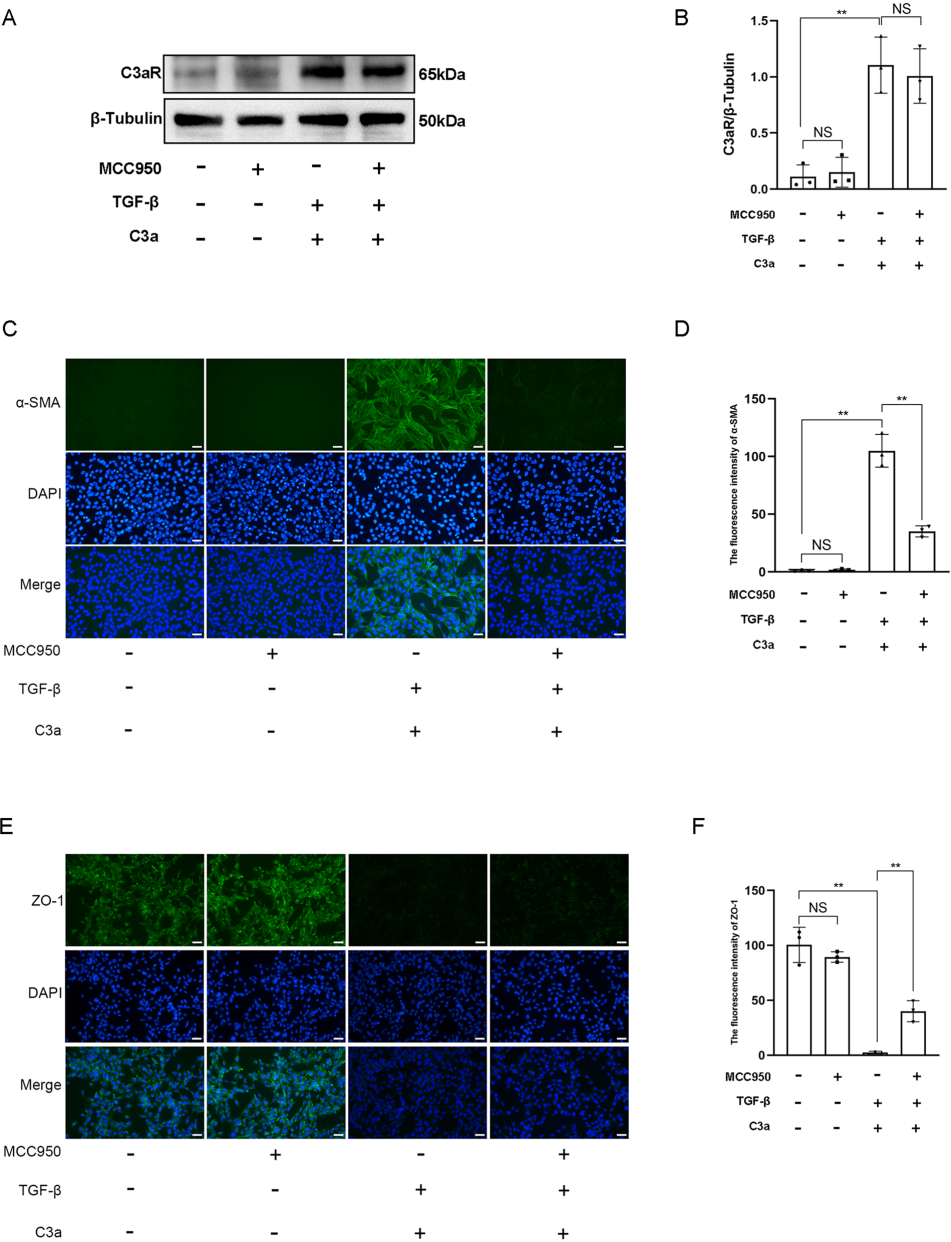
Inhibition of the NLRP3 Inflammasome alleviated EMT but didn’t affect the expression of C3aR. **A** Western blot of C3aR of TCMK-1 cells induced by MCC950, TGF-β + C3a, and TGF-β + C3a + MCC950. **B** Quantitative analysis of C3aR. **C** Immunofluorescent staining of α-SMA of TCMK-1 cells induced by MCC950, TGF-β + C3a, and TGF-β + C3a + MCC950. **D** α-SMA-positive area. **E** Immunofluorescent staining of ZO-1 of TCMK-1 cells induced by MCC950, TGF-β + C3a, and TGF-β + C3a + MCC950. **F** ZO-1-positive area. Scale bar:50 μm.**P < 0.01; NS, No significance

Western blot showed that MCC950 didn’t affect the expression of C3aR induced by C3a combined with TGF-β in TCMK-1 cells (Fig. 6A, B). Nevertheless, immunofluorescent staining showed that MCC950 alleviated EMT induced by C3a combined with TGF-β in TCMK-1 cells (Fig. 6C, D).

### Inhibition of ERK signaling inhibited the activation of the NLRP3 inflammasome

**Fig. 7:**
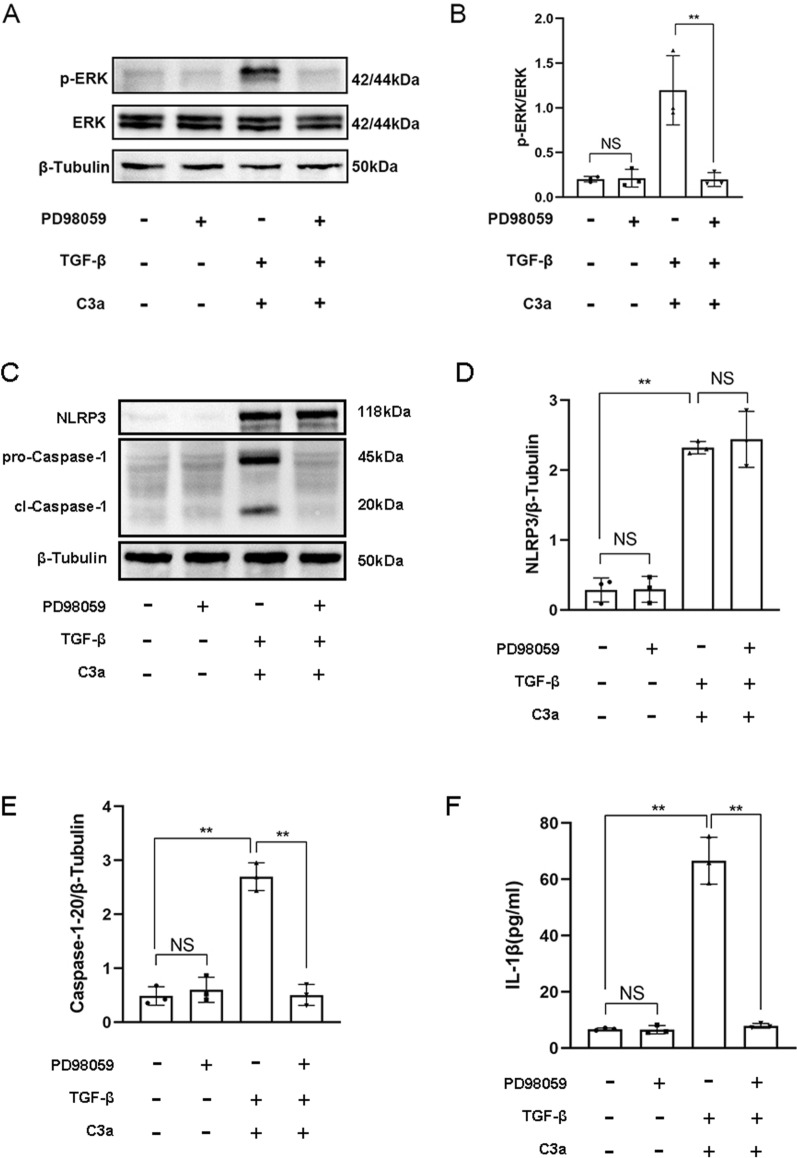
Inhibition of ERK signaling inhibited the activation of the NLRP3 inflammasome. **A** Western blot of p-ERK of TCMK-1 cells induced by PD98059, TGF-β + C3a, and TGF-β + C3a + PD98059. **B** Quantitative analysis of p-ERK. **C** Western blot of NLRP3 and Caspase-1–20 of TCMK-1 cells induced by PD98059, TGF-β + C3a, and TGF-β + C3a + PD98059. **D** Quantitative analysis of NLRP3. **E** Quantitative analysis of Caspase-1–20. **F** ELISA of IL-1β of TCMK-1 cells induced by PD98059, TGF-β + C3a, and TGF-β + C3a + PD98059. **P < 0.01; NS, No significance

The phosphorylation of ERK was activated by C3a combined with TGF-β in TCMK-1 cells, and PD98059, an inhibitor of ERK signaling, inhibited the phosphorylation of ERK significantly (Fig. 7A, B). PD98059 didn’t affect the expression of NLRP3, but it suppressed the expression of Caspase-1–20 and the secretion of IL-1β (Fig. 7C–F). These data suggested that inhibition of ERK signaling inhibited the activation of the NLRP3 inflammasome.

## Discussion

EMT represents a promising therapeutic target for renal interstitial fibrosis, the primary pathological change underlying CKD. Both C3a and the NLRP3 inflammasome have been reported to contribute to EMT [8, 10]. However, the relationship between them in EMT has not been clearly explained. In this study, EMT was induced by C3a combined with TGF-β in TCMK-1 cells. Our findings demonstrated the activation of C3aR and the NLRP3 inflammasome, and the phosphorylation of ERK during EMT. Inhibition of C3aR did not impact the expression of the NLRP3 protein, but attenuated the activation of the NLRP3 inflammasome and ameliorated EMT. Inhibition of the NLRP3 inflammasome also alleviated EMT but didn’t affect the expression of C3aR. Furthermore, inhibition of ERK signaling had no effect on the expression of the NLRP3 protein but diminished the activation of the NLRP3 inflammasome. Therefore, we speculated that C3aR collaborates with TGF-β via the ERK pathway to activate the NLRP3 inflammasome and promote EMT in RTECs. Similar findings were observed in the UUO model, a typical model of renal interstitial fibrosis. Compared to sham-operated mice, the expression of IL-1β in RTECs of UUO mice significantly increased. Knocking out C3aR contributed to lower expression of IL-1β in RTECs of UUO mice. Inhibiting the NLRP3 inflammasome also reduced the expression of IL-1β in RTECs of UUO mice.

EMT is the transdifferentiation of epithelial cells into mesenchymal cells. Epithelial cells are tightly packed and mediated by several cell–cell adhesion complexes, including tight junctions, adherens junctions, and desmosomes. The cells act as a protective barrier against microorganisms, mechanical stress, ultraviolet radiation, and more. However, certain embryonic processes, such as gastrulation, heart development, and neural crest formation, require cell migration. As a result, these cell–cell adhesion complexes disappear [13]. The loss of cell adhesion complexes is commonly accompanied by the up-regulation of several genes related to mesenchymal cell phenotype and down-regulation of those related to epithelial characteristics. For example, E-cadherin and ZO-1(the markers of epithelial cells) decrease, however, α-SMA (the marker of mesenchymal cells) increases [14]. This process is called EMT. In addition to embryonic development, EMT is also involved in fibrosis [15]. In the process of EMT induced by gene mutation or growth factors (such as TGF-β, WNT and Sonic Hedgehog), the cells gradually lose the structure of epithelial cells and switch into a spindle-shaped ones. Meanwhile, their adhesion declines, replaced by strong migration, invasion and anti-apoptosis capabilities, resulting in turning to mesenchymal cells. The expression of transcription factor such as Snail also increases, which initiates and maintains EMT [16, 17]. Early studies on regulatory mechanisms have shown that the transcription factor SNAIL1 can directly bind to the promoter of E-cadherin to inhibit its transcription [18, 19]. Since then, transcription factors such as ZEB2, ZEB1, SNAIL2, TWIST1 and E47 have also been identified as the main drivers of transcriptional program of EMT [14].

In our study, TGF-β and C3a were employed to induce EMT in RTECs (TCMK-1). TGF-β, known as a canonical inducer of EMT, regulates transcription through both SMAD and non-SMAD signaling [20, 21]. SMAD signaling involves the recognition and binding of TGF-β by its type II receptor (TβR II), which recruits and phosphorylates its type I receptor (TβR I), leading to the activation of receptor-SMAD (R-SMAD) complex proteins, which subsequently form a trimeric complex with Co-SMAD/SMAD4 and translocate into the nucleus as transcription factors to regulate target gene expression [22, 23]. Smad2/Smad3 in SMAD signaling are considered pivotal mediators of TGF-β signaling in tissue fibrosis and tumorigenesis, while Smad6 and Smad7 function as negative regulators of fibrosis and tumorigenesis mediated by TGF-β [21]. Non-SMAD signaling encompasses all pathways activated by TGF-β through phosphorylation, acetylation, ubiquitination, sumoylation and protein–protein interactions, along with downstream cascades [24]. Our results also showed that the expression of α-SMA increased and the expression of ZO-1 decreased after TCMK-1 cells were stimulated by TGF-β, suggesting TGF-β is the inducer of EMT.

The complement system is a complex system consisting of over 30 types of soluble proteins, membrane-binding proteins, and complement receptors. It is present in serum and tissue fluid of human and animals. Upon activation, it exerts various biological effects including regulation of phagocytosis, cell lysis, inflammation mediation, immune regulation, and clearance of immune complexes. The complement system can be activated through three pathways: classical pathway, lectin pathway, and bypass pathway [5]. During the process of complement activation, C3 undergoes cleavage into two smaller fragments, C3a and C3b [25, 26]. C3a, known as an anaphylatoxin, can mediate a range of kidney diseases when combining with C3aR. C3a/C3aR-induced EMT has been reported in recent years. C3aR antagonist effectively inhibited C3a-induced EMT, improved renal function, and inhibited renal interstitial fibrosis in mice with adriamycin-induced proteinuric nephropathy [8]. Our previous studies also demonstrated that C3a can induce EMT in renal tubular epithelial cells [6, 7]. However, the underlying mechanism remains incompletely understood. This study revealed that co-intervention of TGF-β and C3a significantly enhanced the effect of EMT compared to their individual interventions. We speculated it might be related to the activation of the NLRP3 inflammasome.

NLRP3 is a member of the nucleotide-binding oligomerized domain-like receptor family. The expression of NLRP3 increases when cells are exposed to pathogen-associated molecular patterns (PAMP) or damage-associated molecular pattern (DAMP) (priming stage) [27]. Then apoptosis-associated specklike protein (ASC) and pro-Caspase-1 are recruited to form the NLRP3 inflammasome and Caspase-1 is activated (inflammasome assembly stage). The active Caspase-1 cleaves pro-IL-1β and pro-IL-18 to produce active IL-1β and IL-18 which promote inflammation, pyroptosis and fibrosis [9]. It is currently controversial about the relationship between TGF-β and the NLRP3 inflammasome in EMT. Some studies suggested that the NLRP3 inflammasome induced EMT through TGF-β. In bleomycin-induced pulmonary fibrosis model, the activation of the NLRP3 inflammasome promotes the expression of TGF-β, resulting in EMT. Silencing NLRP3 reduced TGF-β level reduction, contributing to EMT improvement [28]. The mechanism may be that IL-1β secreted by the activation of the NLRP3 inflammasome binds to its receptor and promotes the transcription of TGF-β through MyD88 and c-JUN. TGF-β binds to TβR II in autocrine and paracrine ways, leading to the phosphorylation of Smad2/3 and combining with Smad4. Finally, the expression of EMT-related genes are promoted [29, 30]. Some studies suggested that TGF-β induces the activation of the NLRP3 inflammasome to promote EMT and fibrosis [10, 11]. H_2_S blocks the activation of the NLRP3 inflammasome by down-regulating TGF-β1 signaling, thus improving vascular fibrosis [31]. Nevertheless, some studies suggested that TGF-β induced the expression of NLRP3 to promote EMT independently of the activation of the NLRP3 inflammasome [32]. Our study revealed that TGF-β alone could only induce the expression of the NLRP3 protein, rather than activate the whole inflammasome. However, with the assistance of C3a, TGF-β could induce the activation of the whole inflammasome. Researches on C3a-induced activation of the NLRP3 inflammasome mainly focus on inflammatory cells [33, 34]. Whether C3a can mediate EMT by activating the NLRP3 inflammasome in RTECs has not been reported. Our study showed that on the premise of TGF-β-induced expression of the NLRP3 protein, C3a/C3aR promoted the assembly of the NLRP3 inflammasome, resulting in the activation of Caspase-1 and the secretion of IL-1β. This suggested that C3aR synergies with TGF-β to activate the NLRP3 inflammasome to promote EMT of RTECs, and the way in which C3aR activates the inflammasome is to promote the assembly of the NLRP3 inflammasome. This viewpoint also confirms the conclusion of our previous study in vivo [12]. Our previous study has concluded that C3aR aggravates renal interstitial fibrosis by regulating the activation of the NLRP3 inflammasome (particularly regulating the assembly of the inflammasome) in UUO mice.

**Fig. 8:**
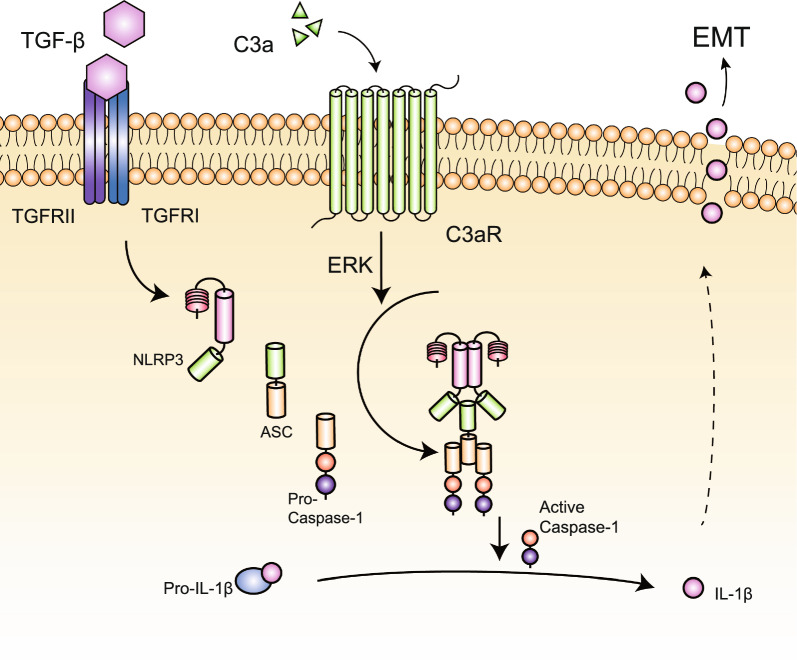
C3a/C3aR synergizes with TGF-β to promote the activation of the NLRP3 inflammasome via ERK signaling, resulting in EMT of renal tubular epithelial cells

It has been reported that C3a/C3aR mediates the assembly of the NLRP3 inflammasome in human monocytes via ERK signaling [33]. We hypothesized that ERK plays a similar role in RTECs, that is, C3aR promotes the assembly of the NLRP3 inflammasome through ERK. We also wanted to verify whether ERK signaling plays a role in the expression of the NLRP3 protein induced by TGF-β. Because it has been reported that TGF-β can regulate tumor growth, invasion and metastasis through ERK [35], and TGF-β/ERK may also be involved in the EMT process of diabetic cardiomyopathy [36]. However, whether TGF-β promotes the expression of  the NLRP3 protein through ERK signaling has not been reported. Our results showed that phosphorylated ERK increased when the NLRP3 inflammasome was activated by C3a combined with TGF-β in renal tubular epithelial cells. Inhibition of ERK phosphorylation by PD98059 did not decrease the expression of the NLRP3 protein, but suppressed the expression of activated Caspase-1 and the secretion of IL-1β. This result suggested that C3a/C3aR mediates the assembly of the NLRP3 inflammasome through ERK signaling, while ERK signaling is not involved in TGF-β-induced expression of the NLRP3 protein. Therefore, we propose that C3aR synergies with TGF-β to activate the NLRP3 inflammasome to promote EMT of RTECs through ERK signaling, and the way in which C3aR activates the NLRP3 inflammasome is to promote the assembly of the NLRP3 inflammasome (Fig. 8).

There are some limitations to this study. Firstly, the role of C3a/C3aR in facilitating the NLRP3 inflammasome assembly through ERK signaling remains largely unexplored. Secondly, the mechanism by which NLRP3 induces EMT was not discussed. We hypothesize that IL-1β produced by the activation of the NLRP3 inflammasome may promote EMT. The activation of the NLRP3 inflammasome may also further enhance EMT promoted by TGF-β. We will explore the relationship between the NLRP3 inflammasome and TGF-β in the future study. Thridly, the relationship between C3aR and the NLRP3 inflammasome was not discussed in CKD specimens. We will collect renal biopsy samples of CKD patients to examine the expressions of C3aR and the NLRP3 inflammasome. Together with other nephropathy indicators such as proteinuria, blood pressure, blood C3 levels and renal pathology, we will further investigate the potential of C3aR and the NLRP3 inflammasome as diagnostic and prognostic markers of CKD.

## Conclusion

Our study demonstrated that C3a/C3aR synergies with TGF-β to activate the NLRP3 inflammasome to promote epithelial-mesenchymal transition of renal tubular epithelial cells through ERK signaling, and the way in which C3aR activates the inflammasome is to promote the assembly of the NLRP3 inflammasome. This will provide some new insights for the new biomarkers and therapeutic targets of epithelial-mesenchymal transition.

## Data Availability

All data generated or analysed during this study are included in this published article.

## Abbreviations

ATCC: American type culture collection
α-SMA: α-Smooth muscle actin
C3a: Complement component 3a
C3aR: Complement component 3a receptor
C3aRa: C3aR antagonist; CKD: chronic kidney disease
DAMP: Damage-associated molecular pattern
DAPI: 4’,6-Diamidino-2-phenylindole
ELISA: Enzyme linked immuno sorbent assay
EMT: Epithelial-mesenchymal transition
ERK: Extracellular regulated protein kinase
IL: Interleukin
KO: Knockout
NLRP3: Nucleotide-binding oligomerization domain-like receptor protein-3
PAMP: Pathogen-associated molecular pattern
PBS: Phosphate-buffered saline
TGF-β: Transforming growth factor-β
UUO: Unilateral ureteral obstruction
WT: Wide type
ZO-1: Zonula occludens-1
